# A long-term cost-effectiveness analysis of cardiac resynchronisation therapy with or without defibrillator based on health claims data

**DOI:** 10.1186/s12962-022-00384-x

**Published:** 2022-09-02

**Authors:** Moritz Hadwiger, Laura Schumann, Nora Eisemann, Nikolaos Dagres, Gerhard Hindricks, Janina Haug, Michael Wolf, Ursula Marschall, Alexander Katalinic, Fabian-Simon Frielitz

**Affiliations:** 1 Institute of Social Medicine and Epidemiology, University of Lübeck, Ratzeburger Allee 160, 23538 Lübeck, Germany; 2 Department of Electrophysiology, Heart Center Leipzig at University of Leipzig, Leipzig, Germany; 3 Leipzig Heart Institute, Russenstraβe 69A, 04289 Leipzig, Germany; 4 The Clinical Research Institute, Munich, Germany; 5 BARMER, Wuppertal, Germany

**Keywords:** Cardiac resynchronisation, Health claims data, Cost-effectiveness analysis

## Abstract

**Background:**

In Germany, CRT devices with defibrillator capability (CRT-D) have become the predominant treatment strategy for patients with heart failure and cardiac dyssynchrony. However, according to current guidelines, most patients would also be eligible for the less expensive CRT pacemaker (CRT-P). We conducted a cost-effectiveness analysis for CRT-P devices compared to CRT-D devices from a German payer’s perspective.

**Methods:**

Longitudinal health claims data from 3569 patients with de novo CRT implantation from 2014 to 2019 were used to parametrise a cohort Markov model. Model outcomes were costs and effectiveness measured in terms of life years. Transition probabilities were derived from multivariable parametric survival regression that controlled for baseline differences of CRT-D and CRT-P patients. Deterministic and probabilistic sensitivity analyses were conducted.

**Results:**

The Markov model predicted a median survival of 84 months for CRT-P patients and 92 months for CRT-D patients. In the base case, CRT-P devices incurred incremental costs of € − 13,093 per patient and 0.30 incremental life years were lost. The ICER was € 43,965 saved per life year lost. In the probabilistic sensitivity analysis, uncertainty regarding the effectiveness was observed but not regarding costs.

**Conclusion:**

This modelling study illustrates the uncertainty of the higher effectiveness of CRT-D devices compared to CRT-P devices. Given the difference in incremental costs between CRT-P and CRT-D treatment, there would be significant potential cost savings to the healthcare system if CRT-D devices were restricted to patients likely to benefit from the additional defibrillator.

**Supplementary Information:**

The online version contains supplementary material available at 10.1186/s12962-022-00384-x.

## Introduction

Heart failure (HF) is a common condition associated with a high hospitalisation rate, reduced longevity and impaired quality of life [1, 2]. Standard management of heart failure due to reduced heart contractility (known as heart failure with reduced ejection fraction) includes medication and, in people with certain features, cardiac electronic implantable devices. For those with evidence of electrical dyssynchrony, which is linked to additional symptom burden, and worse clinical outcomes, a pacemaker device to resynchronise the heart's contraction known as cardiac resynchronisation therapy (CRT), can be implanted. CRT is proven to reduce the severity of heart failure measured according to the New York Heart Association (NYHA) classification and related mortality and hospitalisations [3, 4]. CRT can be delivered by a pacemaker (CRT-P) or in combination with a defibrillator (CRT-D) to provide additional protection from sudden cardiac death (SCD).

For the majority of patients, it is controversial whether a defibrillator is necessary [5]. CRT already inherently reduces the risk of SCD [6]. The implementation of extended [7, 8] and better [9] drug therapy has also influenced SCD rates. According to a review, sudden cardiac death rates in CRT patients with heart failure have continued to decrease over time, and the difference between CRT-D and CRT-P patients has reduced [10]. Moreover, there has never been a sufficiently powered randomised controlled trial (RCT) comparing CRT-P and CRT-D devices. Despite the improved outcomes and reduced sudden death rates, CRT-D devices remain the primary treatment strategy in Germany for various clinical and non-clinical reasons [11]. However, the costs of CRT-D device implantation are nearly 40% higher than CRT-P devices [12].

The *Re-evaluation of optimal re-synchronisation therapy in patients with chronic heart failure* (RESET-CRT) [13] project compares the survival of CRT-P patients to that of CRT-D patients in a still ongoing RCT. It is so far hypothesised that CRT-P is non-inferior to CRT-D regarding survival. Additionally to the ongoing randomised trial, results of a survival analysis of patients with de novo CRT-P and CRT-D implantation based on German health claims data from 2014 to 2019 that were recently published showed no significant survival difference between CRT-P and CRT-D patients after correcting for confounders [14].

This study uses said health claims dataset to assess the long-term cost-effectiveness of CRT-P devices compared to CRT-D devices from a German payer’s perspective by extrapolating the clinical outcomes in a cohort Markov model. Model outcomes were life years and costs from a payer’s perspective.

## Data and methods

### Data source

The authors initially prepared the dataset for a survival analysis from health claims data [14]. The Markov model was parameterised using routinely collected health claims data from the BARMER, a large nationwide German statutory health insurance (SHI), which insures 8.9 million individuals [15]. Health insurance is mandatory in Germany, and approximately 90% of the population is insured in statutory health insurance (SHI) [16]. The database contains longitudinal patient-level data on inpatient and outpatient utilisation in terms of related costs, socio-demographics, and all-cause deaths from 2005 to 2019.

### Study population

All patients with a CRT implantation from 2014 to 2019 in the BARMER database were considered for study inclusion (N = 7082). Detailed information on the patient population selection can be found elsewhere [14]. In brief, patients had to be older than 18 years and have symptomatic heart failure with de novo CRT implantation. Exclusion criteria were an indication for implantation of a cardioverter defibrillator for secondary prevention, acute coronary syndrome, cardiac revascularisation therapy, cardiac valve surgery, or a previous percutaneous cardiac valvular intervention. The International Classification of Diseases (ICD) codes and Operation and Procedure Codes (OPS) were used for the inclusion and exclusion of patients. ICD codes were used to identify diagnoses, and OPS codes were used to identify procedures such as CRT implantation. Patients needed to have been observed 3 years before implantation and over a follow-up period of at least three months after CRT implantation or died during this time; otherwise, they were excluded. After applying the inclusion and exclusion criteria, 3569 patients with CRT de novo implantations were included in the analysis. Of these, 847 were CRT-P implantations, and 2722 were CRT-D implantations.

### Markov model

**Fig. 1:**
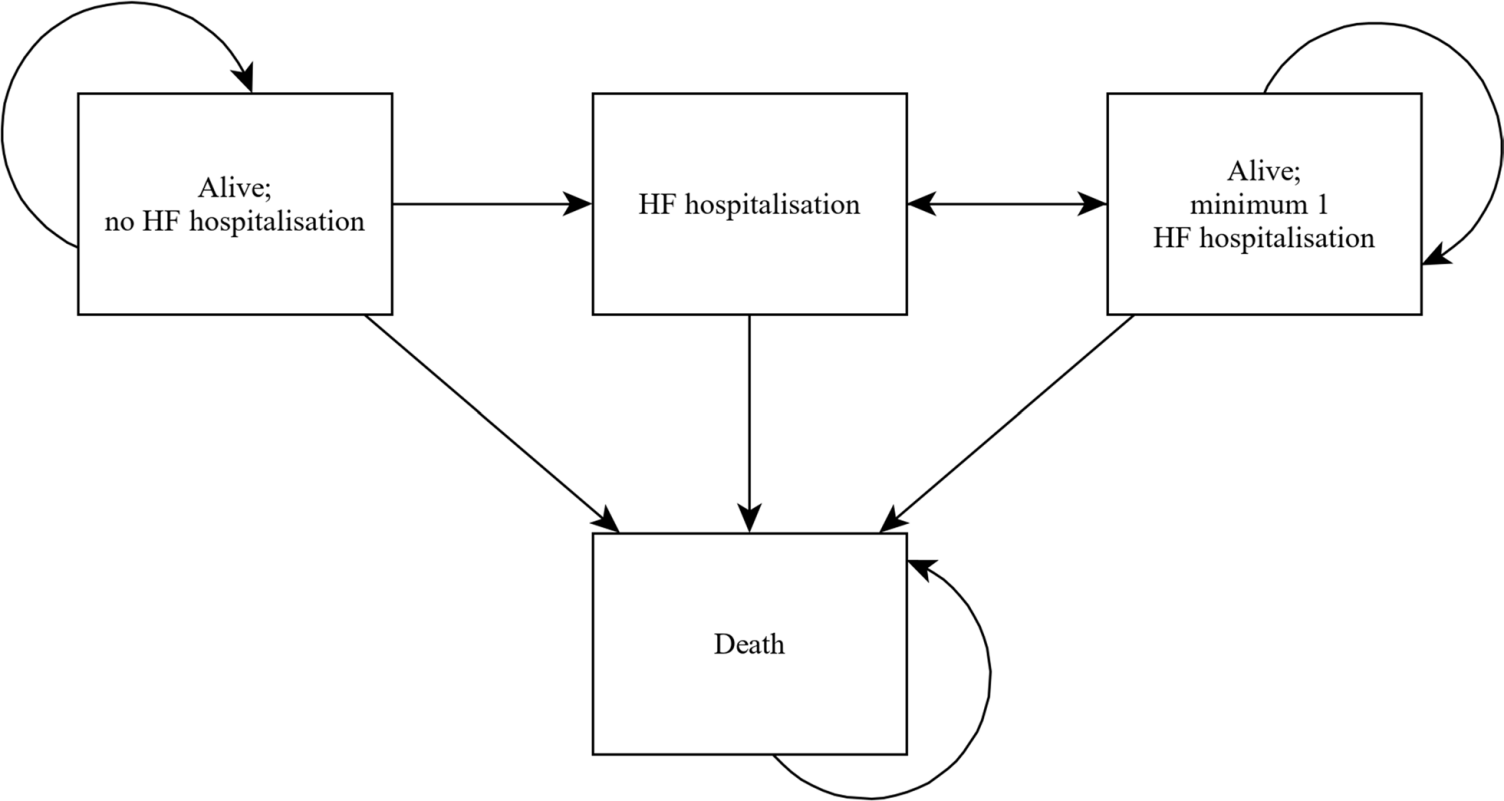
Markov model structure. HF: heart failure

The model consists of four Markov-states (Fig. 1), namely “Alive; no HF hospitalisation”, “Alive; at least 1 HF hospitalisation”, and “Month with HF hospitalisation” as a tunnel state dividing the previous two states and “Death” as the absorbing state. The states of subsequent hospitalisation for heart failure after CRT implantation reflect the disease’s progressive nature.

The modelling guideline recommends a lifetime model for chronic diseases such as heart failure [17]. We limited our model time horizon to 15 years because (1) 90% of the model population died within that time frame, (2) the major health economic outcomes were already captured, and (3) a longer time horizon would have added unnecessary uncertainty. Model results for the maximum follow-up of 6 years and a time horizon of 10 years were reported. The cycle length of the Markov model was one month. Model outcomes were life years and costs. According to the German guideline, both outcomes were discounted by 3% per year [18]. The device-specific differences in hazard ratios converged after the observation period (6 years); after ten years, the hazard ratio (HR) had to take a value of 1 to reflect that treatment effects diminish over time. The analysis was conducted with “R” (R Foundation for Statistical Computing, Vienna, Austria) [19].

### Parametrisation

#### Effectiveness

Effectiveness was measured by life years (survival of CRT-P patients vs survival of CRT-D patients). The index date was defined as the date of CRT device implantation. The follow-up time was defined as time between the CRT implantation and death or censoring. The follow-up time was censored if the patient was still alive after the end of the observation period, i.e. 2019-12-31, or if the patient left BARMER for other reasons. In addition to the time-to-death, we considered the time-to-hospitalisation due to heart failure. During follow-up, 714 deaths were observed, and 843 patients experienced 1627 hospitalisations for heart failure.

To account for the observational character of our data, we used several control variables. We included the demographic characteristics age and sex, and the comorbidities renal dysfunction (stage III, IV), diabetes, and atrial fibrillation. In addition, we considered the aetiology of heart failure (ischemic/non-ischemic) and the number of hospitalisations before 1 year to implantation (0, 1, 2, > 2).

Transition probabilities were estimated using age-dependent multivariable parametric survival regressions. The process of deriving those transition probabilities was structured in three steps. First, we estimated multivariable parametric survival models for exponential, Weibull, Gompertz, log-logistic, and lognormal distributions and extracted the regression coefficients. Second, for each parametric survival distribution, we computed age-dependent survival curves for our cohort simulation patients for CRT-D and CRT-P devices. The cohort simulation patients represented the average baseline characteristics of CRT-D patients because the study’s main interest was how CRT-D patients would perform if treated with CRT-P devices. Third, we plotted the estimated parametric survival curves for CRT-D patients in comparison to the observed CRT-D Kaplan–Meier curves (Additional file 1: Figs. S2–S5). The selection of the appropriate parametric survival function was aided by a review of publications and a guide on survival analysis for modelling studies [20, 21]. We selected the parametric survival distribution with good visual conformity to the CRT-D Kaplan–Meier curve for the first 6 years and with a plausible shape in the long run (plausibility was given when the probability of death did not decrease with increasing age).

**Fig. 2:**
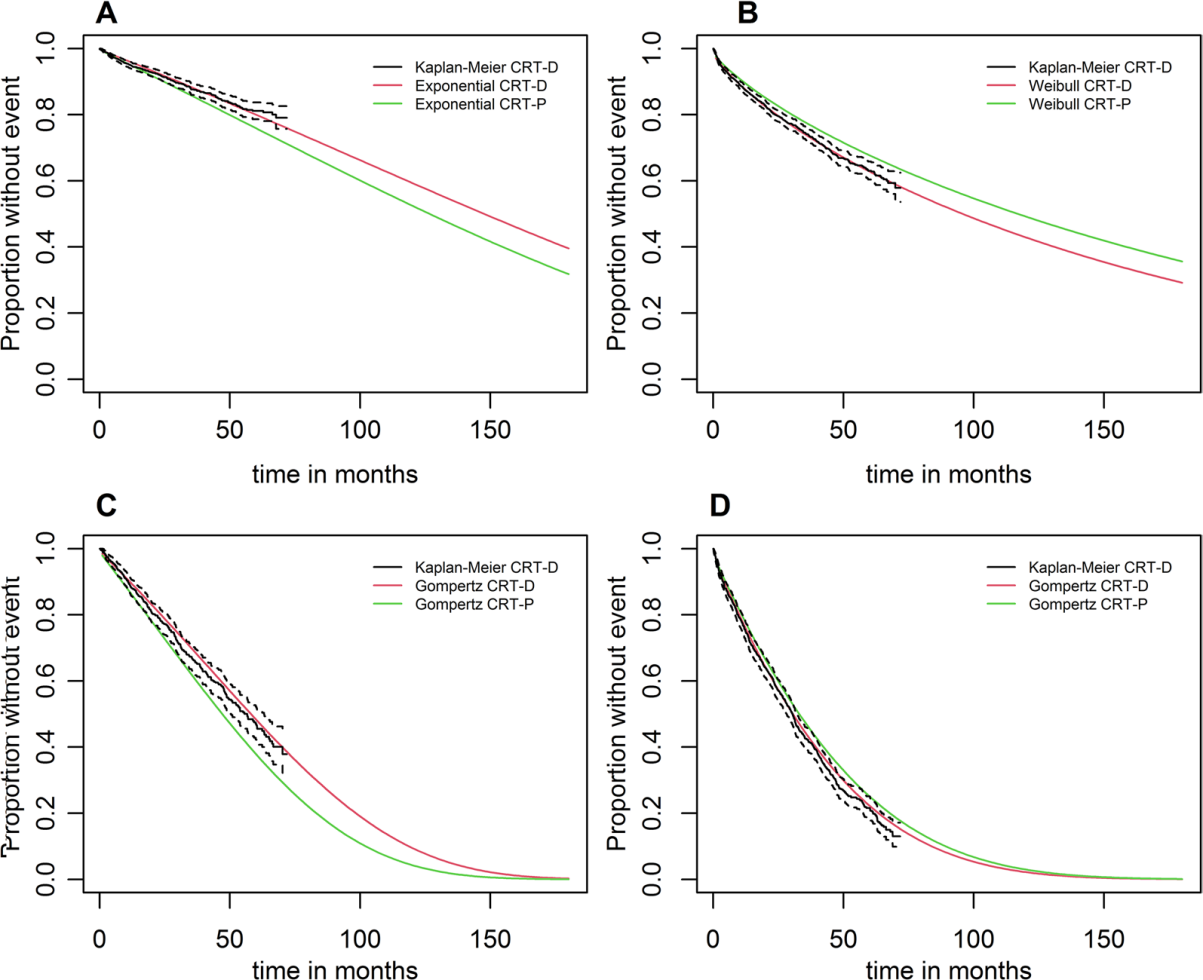
**A**–**D** Parametric survival curves fitted to Kaplan-Meier curves; **A** Survival without subsequent HF hospitalisation. **B** First HF Hospitalisation. **C** Survival with subsequent HF hospitalisation. **D** Further HF hospitalisations. CRT-P: cardiac biventricular pacemaker, CRT-D: cardiac biventricular defibrillator, HF: heart failure

An exponential distribution was chosen to transition from the state “Alive; no HF hospitalisation” to the state “Death”. For the transition from “Alive; no HF hospitalisation” to “Month with HF hospitalisation”, a Weibull distribution was chosen. A Gompertz distribution was selected for the transition from “Alive; at least 1 HF hospitalisation” to “Death” and for the transition from “Alive; at least 1 HF hospitalisation” to “Month with HF hospitalisation” The plot of the parametric survival curves chosen can be found in Fig. 2A–D and the regression results in Additional file 1: Tables S1–S4. To check the proportional hazard assumption, log-cumulative hazard graphs were plotted for each variable and were found to be reasonably parallel.

#### Cost data

We calculated mean direct costs based on claims data from 2014 to 2019 for all patients. Costs were inflated to 2019 prices [22]. We excluded the highest 5% of the observations to limit distortion due to outliers. The cost for CRT implantation includes fees for the device itself as well as the inpatient treatment costs. The implantation costs were considered device-dependent, but hospitalisation costs due to heart failure, outpatient costs and medication costs were not. Medication in the anatomical-therapeutic-chemical category for cardiovascular systems was considered to reflect the cost of medications related to heart failure [23]. Costs for device replacements were calculated from all device changes in the BARMER database in 2019. Device runtimes of CRT-P and CRT-D devices were obtained from a separate analysis of the BARMER database [24]. The median runtime was 8.16 years for CRT-P devices and 6.04 years for CRT-D devices. Future medical costs, defined as healthcare expenditures excluding heart failure costs, were considered using the data of Gandjour and Ostwald (2018) [25]. Information on costs and CRT device runtime are displayed in Table 1.

**Table 1:** Costs and CRT device runtime

Input parameter	Mean	SD	PSA distribution
*Costs in €*			
Implantation CRT-P	11,092.44	2807.39	Gamma
Implantation CRT-D	16,648.03	2857.33	Gamma
Replacement CRT-P	5643.52	1209.20	Gamma
Replacement CRT-D	8095.18	1123.75	Gamma
Heart failure hospitalisation	4077.23	2611.73	Gamma
Quarterly outpatient costs	49.25	47.98	Gamma
Monthly medication costs	180.82	205.54	Gamma
Future medical costs unrelated to HF (65–84 years)	7275	–	–
Future medical costs unrelated to HF (older 85 years)	16,616	–	–
*Device longevity in months*			
CRT-P	98*	–	Poisson
CRT-D	72*	–	Poisson

CRT-P: cardiac biventricular pacemaker, CRT-D: cardiac biventricular defibrillator, HF: heart failure, SD: standard deviation, PSA: probabilistic sensitivity analysis

*Median

### Sensitivity analyses

Patients with two different consecutive NYHA class codes before CRT implantation (N = 141) were included in a sensitivity analysis with their higher NYHA class.

Additionally, a deterministic sensitivity analysis (DSA; input parameters are given in Additional file 1: Table S5) and a probabilistic sensitivity analysis of the base case (PSA; a Monte Carlo simulation with 10,000 runs) were conducted. While the DSA was applied to assess the effect of variations of individual parameters on the model results, the PSA aimed at estimating the effect of global uncertainty on the model results. The PSA took into account the correlation between the predictors of the survival regression models by using a Cholesky decomposition of the covariance matrix [26]. Probability distributions for all other input parameters were chosen according to the recommendations of the ISPOR guideline [27].

## Results

### Base case

Baseline characteristics of the study sample are provided in Table 2. The starting age of the Markov model cohort was 69.9 years, which equals the average age of CRT-D patients in the dataset. The model predicted median survival of 84 months for CRT-P patients and 92 months for CRT-D patients. After six years, the model predicted the survival of 63% of the CRT-D patients, comparable to the observed survival of CRT-D patients (65%; 95% confidence interval: 62%–68%). Overall, the modelled CRT-D survival matched the observed CRT-D survival quite well, and the differences were small (Additional file 1: Table S6). The average number of hospitalisations for heart failure predicted by the model was 1.36 for CRT-P patients and 1.43 for CRT-D patients. In the base case (15 years), the average treatment costs were € 81,241 for CRT-P patients and € 94,335 for CRT-D patients, resulting in negative incremental costs of € − 13,093 per patient. Over the time horizon of 15 years, 0.30 life years were lost. In this case, the incremental cost-effectiveness ratio (ICER) indicated the costs saved per life year lost. The ICER for CRT-P compared to CRT-D was € 43,965. Table 3 shows the results for the other model time horizons. After a model horizon of 10 years, the ICER per life year lost was € 40,824, and after the maximum follow-up of 6 years, the ICER amounted to € 77,865.

**Table 2:** Baseline characteristics of patients at CRT implantation

Variables	CRT-D	CRT-P
Number of observations	2722	847
Male sex, *No.* (%)	1768 (65)	440 (52)
Age, mean (SD), years	69.95 (9.57)	76.65 (8.89)
Non-ischemic, *No.* (%)	678 (25)	225 (27)
*Hospital visits 1 year before implantation, No. (%)*		
0	121 (4)	40 (5)
1	846 (31)	274 (32)
2	868 (32)	222 (26)
> 2	887 (33)	311 (37)
Diabetes, *No.* (%)	982 (36)	272 (32)
Renal dysfunction stage 3, *No.* (%)	749 (28)	300 (35)
Renal dysfunction stage 4, *No.* (%)	112 (4)	58 (7)
Atrial fibrillation, *No.* (%)	1105 (41)	497 (59)

CRT-P: cardiac biventricular pacemaker; CRT-D: cardiac biventricular defibrillator; NYHA: New York Heart Association; SD: standard deviation

**Table 3:** Model results for CRT-P device treatment compared to CRT-D device treatment for 15 years, 10 years, and maximum of follow-up (6 years)

Model results	Variable	CRT-P	CRT-D	Difference
Base case: Extrapolation to 15 years	Costs (€)	81,241.24	94,334.54	− 13,093.3
	Life Years	6.55	6.85	− 0.30
	ICER			43,964.91
Extrapolation to 10 years	Costs (€)	72,870.11	84,609.29	− 11,739.18
	Life Years	5.86	6.15	− 0.29
	ICER			40,823.75
Maximum follow-up (6 years)	Costs (€)	56,764,79	68,728.73	− 11,693.94
	Life years	4.49	4.64	− 0.15
	ICER			77,865.49

CRT-P: cardiac biventricular pacemaker, CRT-D: cardiac biventricular defibrillator, ICER: incremental cost-effectiveness ratio

### Sensitivity analyses

The sensitivity analysis, including patients with ambiguous NYHA coding, yielded 0.34 life years lost and a negative incremental cost of € − 14,469. The ICER was € 42,925.

**Fig. 3:**
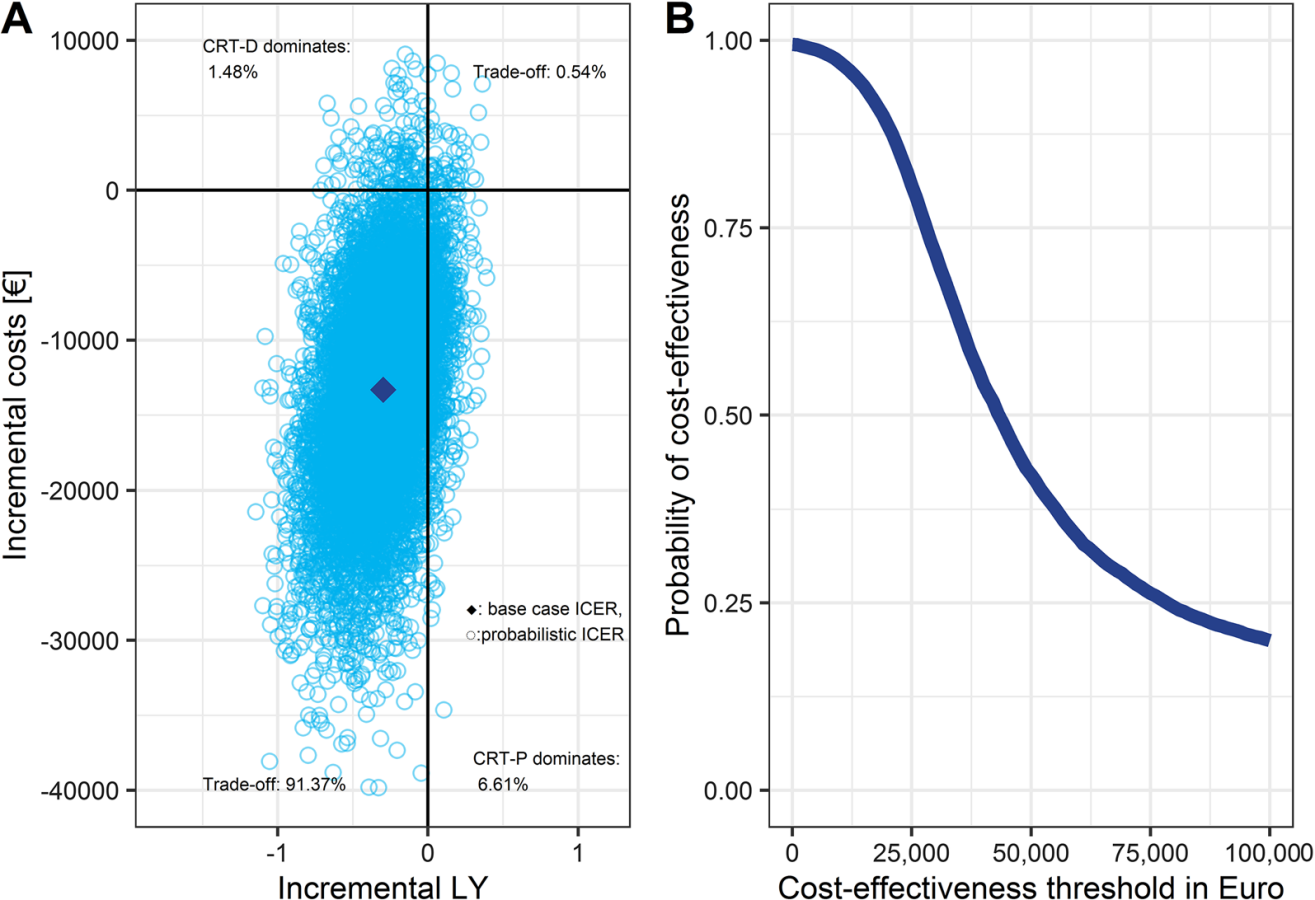
**A** Monte Carlo simulation Probabilistic sensitivity analysis CRT-P vs. CRT-D: results of 10,000 model iterations (Monte Carlo simulation). The scatterplot depicts uncertainty in the model regarding costs and life years for CRT-P patients relative to CRT-D patients. **B** Cost-effectiveness acceptability (CEAC) curve CRT-P vs. CRT-D. The CEAC illustrated the proportion of ICERs from the Monte Carlo simulation that was above the chosen willingness to accept a negative outcome. CRT-P: cardiac biventricular pacemaker, CRT-D: cardiac biventricular defibrillator, ICER: incremental cost-effectiveness ratio, LY: life year

The deterministic sensitivity analysis showed that the base case result is strongly affected by the hazard ratio of CRT-P compared to CRT-D for the transition from the state “Alive; no HF hospitalisation” to the state “Death”. Other influential parameters were the hazard ratio for the first heart failure hospitalisation and death after a heart failure hospitalisation (results of the DSA are given in Additional file 1: Fig. S6). In addition to the base case and the DSA, we conducted a probabilistic sensitivity analysis for the time horizon of 15 years. In the Monte Carlo simulation, 9137 out of 10,000 iterations (91.37%) were located in the southwest quadrant: a treatment with CRT-P was less effective but less costly. In another 6.61% of the iterations, CRT-P dominated CRT-D, i.e. it was more effective and less expensive. The average ICER of the Monte Carlo simulation was € 41,641 per life year lost (Fig. 3A). The cost-effectiveness acceptability curve (CEAC, Fig. 3B) is inverse to the traditional CEAC because most of our probabilistic sensitivity analysis outcomes are in the southwest quadrant [28]. Therefore, ICER values below the cost-effectiveness threshold are considered cost-effective for a given willingness-to-lose, which implies an obligation to save on the part of the payer. Thus, the cost-effectiveness probability decreases as the savings requirement per lost LY increases.

Moreover, quality-adjusted life years (QALY) were used as an outcome parameter. As no Germany-specific utility values across all NYHA classes are known, the utility estimates of a recent sacubitril/valsartan evaluation were used [29]. The baseline utility value was 0.72, and a disutility of − 0.08 was assigned in the case of hospitalisation. The ICER was € 66,218 savings per QALY lost.

## Discussion

Based on German health claims data, this study analysed the cost-effectiveness of CRT-P devices compared to CRT-D devices from a statutory health insurance perspective. Our base case results suggest a small and uncertain survival benefit of CRT-D over CRT-P, yet at a considerable cost of € 13,093. The ICER was € 43,965 savings per life year lost. The probabilistic sensitivity analysis indicated uncertainty about the effectiveness but not regarding costs.

The effectiveness of CRT-D may not be superior to CRT-P due to recent improvements in pharmacological therapy [30] and because of a general reduction of SCD [31] since the introduction of RCTs [3, 4] as a landmark in this field. A previously published survival analysis using the same dataset found no survival difference between CRT-P and CRT-D patients after adjusting for confounders [14]. Therapy with CRT-P devices caused fewer costs in our main analysis, which was consistent with our probabilistic sensitivity analysis. In most simulations (97.98%), the treatment with CRT-D devices was more costly, which could be attributed to higher device costs and shorter device longevity [32].

According to a review, all other modelling cost-effectiveness studies evaluated CRT-D compared to CRT-P. It reports that ICER range from €7375 to €46,890 in 2014 prices [33]. We compared CRT-P devices to CRT-D devices because CRT-D devices are the predominant treatment strategy in Germany. Our estimated ICER (€ 43,965) fits in the range of the previously mentioned review. In other studies, the estimated incremental effectiveness of CRT-D devices compared to CRT-P devices ranged from 0.65 to 1.69 life years gained, and the incremental cost difference from € 30,879 to € 48,076 [34–38]. Regarding these two outcomes, our results are less extreme.

Moreover, as the follow-up data were limited to 6 years, we had to make assumptions about the later survival benefit of CRT-D patients compared to CRT-P patients. We assumed that the survival benefit of CRT-D starts to fade six years after implantation, with no remaining survival benefit after ten years. This leads to more conservative estimations of the ICER.

The uncertainty about the survival benefit in our probabilistic sensitivity analysis fits the inconsistent results of previous observational studies: analyses of the National Health Service Hospital Episode Statistics reported a survival benefit of CRT-D [39, 40]. Other studies found no survival benefit in non-ischaemic patients [41], older patients [42, 43], patients who survived the first 5 years after CRT implantation [44] and patients with non-ischemic dilated cardiomyopathy [45]. No differences in survival were found in the overall sample in a post hoc analysis of the randomised COMPANION trial [46].

In our modelling approach, survival depends more on age, comorbidities and prevention of further hospitalisations for heart failure after CRT implantation than on device selection (Additional file 1: Tables S1, S2). Therefore, the choice of the CRT device should be based on whether the patient would benefit from an additional defibrillator given the patient’s characteristics [47, 48], which could be assessed, for example, by a risk score. The findings of Barra et al. suggest that the Goldberg risk score may help to discriminate between patients who are likely to benefit from an additional defibrillator and those who are not. A Goldberg score of ≥ 3 might indicate that a patient is unlikely to benefit from an additional defibrillator [49]. A rough calculation suggest that better risk stratification could reduce the proportion of CRT-D implantation from approx. 60% to 20%, leading to a reduction of costs related to CRT implantations in the statutory health insurance of 25.5 million €. This is particularly important in view of other disadvantages of CRT-D device therapy: CRT-D devices are more likely to cause infections [50], device runtime is shorter [24], and device replacements are again associated with a higher risk of infections [51]. In addition, patients suffer from a reduced quality of life due to inappropriate shocks released by the defibrillator [52].

Our analysis has multiple strengths. First, the data used to parameterise the Markov model included more patients and had a longer follow-up period than other CRT modelling studies. Second, the chosen period reflects contemporary medical therapy. Third, we used multivariable age-dependent survival analysis to derive the transition probabilities and to extrapolate beyond the observation period. Fourth, disease progression was modelled by hospitalisation for heart failure, a conventional approach [53]. This choice was supported by a stratified analysis of our dataset, which showed that the probability of death after CRT implantation strongly depended on the first hospitalisation for heart failure after CRT implantation (Additional file 1: Fig. S1). Other modelling approaches use NYHA classes, which are a more subjective measure [54].

This study’s limitations are mainly due to the characteristics of health claims data. First, there is a lack of information on critical clinical parameters for survival, such as QRS duration, left ventricular ejection fraction, or left bundle branch block. Second, the assignment of the CRT device was not randomised. In Germany, CRT-P devices are more commonly used in elderly patients with atrial fibrillation, mild LVSD and comorbidities. For this reason, we used several risk adjustment variables to control for selection bias, but internal validity would be stronger with a randomised assignment to therapy arms, but health claims data are considered to have higher external validity than RCT data [55].

## Conclusion

The results of our modelling approach illustrate the uncertainty of the survival benefit of CRT-D devices compared to CRT-P devices. Consequently, when selecting a CRT device, more attention should be paid to which patients are likely to benefit from the additional defibrillator. This could be achieved using existing risk scores that predict the need for a defibrillator. If CRT-D devices were used more selectively, the overall cost would be lower, and the ICER for CRT-D would be considerably better in this pre-selected group of patients, as the life gain from prescribing CRT-D would be greater. The results highlight the relevance of further investigating survival with CRT-P and CRT-D in an RCT.

## Supplementary Information

**Additional file 1.** A long-term cost-effectiveness analysis of cardiac resynchronisation therapy with or without defibrillator based on health claims data.

## Data Availability

The data that support the findings of this study are owned by the BARMER (Wuppertal, Germany) and are not publicly available.
