# Supplementary material for: A long-term cost-effectiveness analysis of cardiac resynchronisation therapy with or without defibrillator based on health claims data
Source: Cost Eff Resour Alloc. 2022 Sep 2;20:48. doi: 10.1186/s12962-022-00384-x (PMC9438143; doi:10.1186/s12962-022-00384-x)
Supplement: Supplementary file 1 — Additional file 1. A long-term cost-effectiveness analysis of cardiac resynchronisation therapy with or without defibrillator based on health claims data. [file 12962_2022_384_MOESM1_ESM.docx]

# Supplementary Information:

# A long-term cost-effectiveness analysis of cardiac resynchronisation therapy with or without defibrillator based on health claims data

Moritz Hadwiger^1*^, Laura Schumann^1^, Nora Eisemann^1^, Nikolaos Dagres^2,3^, Gerhard Hindricks^2,3^, Janina Haug^4^, Michael Wolf^4^, Ursula Marschall^5^, Alexander Katalinic^1^_,_ Fabian-Simon Frielitz^1^

^1^ Institute of Social Medicine and Epidemiology, University of Lübeck, Ratzeburger Allee 160, 23538 Lübeck, Germany

^2^ Department of Electrophysiology, Heart Center Leipzig at University of Leipzig, Leipzig, Germany

^3^ Leipzig Heart Institute, Russenstraβe 69A, 04289 Leipzig, Germany

^4^ The Clinical Research Institute, Munich, Germany

^5^ BARMER, Wuppertal, Germany

*corresponding author: e-mail address: moritz.hadwiger@uksh.de; telephone number: 0049-451-500-51236

# Supplementary information

## Tables

Table SI1: Regression results for death without a heart failure hospitalisation after CRT implantation (exponential model)

| *Variables* | *Estimate* | *Lower 95* | *Upper 95* |
| --- | --- | --- | --- |
| Rate | (1.04e-05) | (2.69e-06) | (3.99e-05) |
|  | *HR* | *Lower 95%* | *Upper 95%* |
| Device, CRT-P | 1.24 | 0.98 | 1.58 |
| Sex, (ref. female) | 1.72 | 1.35 | 2.19 |
| Age, in years | 1.06 | 1.05 | 1.08 |
| Non-ischemic (ref. ischemic) | 0.96 | 0.72 | 1.27 |
| *Hospital visits 1 year before implantation (reference 0 hospitalisations)* | | | |
| 1 | 1.79 | 0.83 | 3.87 |
| 2 | 2.05 | 0.95 | 4.42 |
| >2 | 2.72 | 1.27 | 5.85 |
| Diabetes (ref. no diabetes) | 1.46 | 1.18 | 1.82 |
| Renal dysfunction stage 3 (ref. no rn^1^) | 1.06 | 0.83 | 1.35 |
| Renal dysfunction stage 4 (ref. no rn^1^) | 2.43 | 1.68 | 3.51 |
| Atrial fibrillation (ref. no atrial fibrillation) | 1.36 | 1.09 | 1.70 |

Hazard ratios were reported; Abbreviations: CRT-P = cardiac biventricular pacemaker; CRT-D = cardiac biventricular defibrillator; ^1^ rn= renal dysfunction

Table SI2: Regression results for death with a heart failure hospitalisation after CRT implantation (Gompertz model)

| *Variables* | *Estimate* | *Lower 95%* | *Upper 95%* |
| --- | --- | --- | --- |
| Shape | 0.01147 | 0.00546 | 0.01748 |
| Rate | 0.00709 | 0.00021 | 0.00240 |
|  | *HR* | *Lower 95%* | *Upper 95%* |
| Device, CRT-P | 1.34 | 1.03 | 1.73 |
| Sex, (ref. female) | 1.33 | 1.05 | 1.68 |
| Age, in years | 1.03 | 1.01 | 1.04 |
| Non-ischemic (ref. ischemic) | 1.16 | 0.88 | 1.53 |
| *Hospital visits 1 year before implantation (reference 0 hospitalisations)* | | | |
| 1 | 1.13 | 0.57 | 2.26 |
| 2 | 1.10 | 0.55 | 2.19 |
| >2 | 1.40 | 0.70 | 2.77 |
| Diabetes (ref. no diabetes) | 1.15 | 0.93 | 1.41 |
| Renal dysfunction stage 3 (ref. no rn^1^) | 1.39 | 1.11 | 1.75 |
| Renal dysfunction stage 4 (ref. no rn^1^) | 1.87 | 1.29 | 2.72 |
| Atrial fibrillation (ref. no atrial fibrillation) | 1.00 | 0.81 | 1.24 |

Hazard ratios were reported; Abbreviations: CRT-P = cardiac biventricular pacemaker; CRT-D = cardiac biventricular defibrillator, ^1^ rn= renal dysfunction

Table SI3: Regression results for the first heart failure hospitalisation after CRT implantation (Weibull model)

| *Variables* | *Estimate* | *Lower 95* | *Upper 95* |
| --- | --- | --- | --- |
| Shape | 0.79689 | 0.75132 | 0.84522 |
| Scale | 0.00176 | 0.00082 | 0.00377 |
|  | *HR* | *Lower 95* | *Upper 95* |
| Device, CRT-P | 0.84 | 0.71 | 1.00 |
| Sex, (ref. female) | 1.13 | 0.97 | 1.30 |
| Age, in years | 1.02 | 1.01 | 1.03 |
| Non-ischemic (ref. ischemic) | 0.87 | 0.73 | 1.04 |
| *Hospital visits 1 year before implantation (reference 0 hospitalisations)* | | | |
| 1 | 1.14 | 0.76 | 1.72 |
| 2 | 1.31 | 0.87 | 1.96 |
| >2 | 1.49 | 1.00 | 2.24 |
| Diabetes (ref. no diabetes) | 1.32 | 1.14 | 1.51 |
| Renal dysfunction stage 3 (ref. no rn^1^) | 1.52 | 1.31 | 1.77 |
| Renal dysfunction stage 4 (ref. no rn^1^) | 2.11 | 1.62 | 2.74 |
| Atrial fibrillation (ref. no atrial fibrillation) | 1.33 | 1.16 | 1.54 |

Hazard ratios were reported; Abbreviations: CRT-P = cardiac biventricular pacemaker; CRT-D = cardiac biventricular defibrillator, ^1^ rn= renal dysfunction

Table SI4: Regression results for further heart failure hospitalisations after the first heart failure hospitalisation (Gompertz model)

| *Variables* | *Estimate* | *Lower 95* | *Upper 95* |
| --- | --- | --- | --- |
| Shape | 0.00688 | 0.00250 | 0.01126 |
| Rate | 0.00511 | 0.00210 | 0.01238 |
|  | *HR* | *Lower 95* | *Upper 95* |
| Device, CRT-P | 0.92 | 0.75 | 1.14 |
| Sex, (ref. female) | 1.24 | 1.05 | 1.46 |
| Age, in years | 1.01 | 1.00 | 1.02 |
| Non-ischemic (ref. ischemic) | 1.07 | 0.88 | 1.30 |
| *Hospital visits 1 year before implantation (reference 0 hospitalisations)* | | | |
| 1 | 1.77 | 0.98 | 3.17 |
| 2 | 1.61 | 0.90 | 2.88 |
| >2 | 1.81 | 1.01 | 3.25 |
| Diabetes (ref. no diabetes) | 1.21 | 1.05 | 1.40 |
| Renal dysfunction stage 3 (ref. no rn^1^) | 1.33 | 1.14 | 1.55 |
| Renal dysfunction stage 4 (ref. no rn^1^) | 1.44 | 1.08 | 1.92 |
| Atrial fibrillation (ref. no atrial fibrillation) | 1.02 | 0.88 | 1.18 |

Hazard ratios were reported; Abbreviations: CRT-P = cardiac biventricular pacemaker; CRT-D = cardiac biventricular defibrillator, ^1^ rn= renal dysfunction

Table SI5: Input parameter for deterministic sensitivity analysis

| *Variable* | *Baseline Value* | | *DSA values* | |
| --- | --- | --- | --- | --- |
|  |  | *lower* | | *upper* |
| Discount rate | 3 % | 0 % | | 5 % |
| Cost HF hospitalisation | 4,077 | 1,465 | | 6,689 |
| Cost CRT-D implantation | 16,648 | 13,791 | | 19,505 |
| Cost CRT-P implantation | 11,092 | 8,285 | | 13,889 |
| Cost CRT-D replacement | 8,095 | 6,971 | | 9,219 |
| Cost CRT-P replacement | 5,644 | 4,434 | | 6,853 |
| Cost ambulant care (per quartal) | 49 | 1 | | 97 |
| Cost medication | 181 | 0 | | 386 |
| Device runtime CRT-D | 72 | 72 | | 73 |
| Device runtime CRT-P | 98 | 95 | | 103 |
| HR death without HF hospitalisation | 1.24 | 0.98 | | 1.58 |
| HR death after HF hospitalisation | 1.34 | 1.03 | | 1.73 |
| HR first HF hospitalisation | 0.84 | 0.71 | | 1.00 |
| HR further HF hospitalisation | 0.92 | 0.75 | | 1.14 |

Abbreviations: CRT-P = cardiac biventricular pacemaker; CRT-D = cardiac biventricular defibrillator, DSA = deterministic sensitivity analysis, HF = heart failure, HR = hazard ratio

Table SI6: Observed CRT-D survival and model predicted CRT-D survival

| *Time in month* | *Survival CRT-D (Kaplan-Meier curve)* | *Survival CRT-D (model)* | *Survival CRT-P (model)* |
| --- | --- | --- | --- |
| 6 | 0.97 | 0.98 | 0.98 |
| 12 | 0.93 | 0.96 | 0.95 |
| 24 | 0.88 | 0.91 | 0.89 |
| 36 | 0.82 | 0.85 | 0.82 |
| 48 | 0.76 | 0.78 | 0.75 |
| 60 | 0.70 | 0.71 | 0.66 |
| 71 | 0.65 | 0.63 | 0.59 |
| 120 | - | 0.33 | 0.31 |
| 180 | - | 0.11 | 0.11 |

Abbreviations: CRT-P = cardiac biventricular pacemaker; CRT-D = cardiac biventricular defibrillator

## Figures


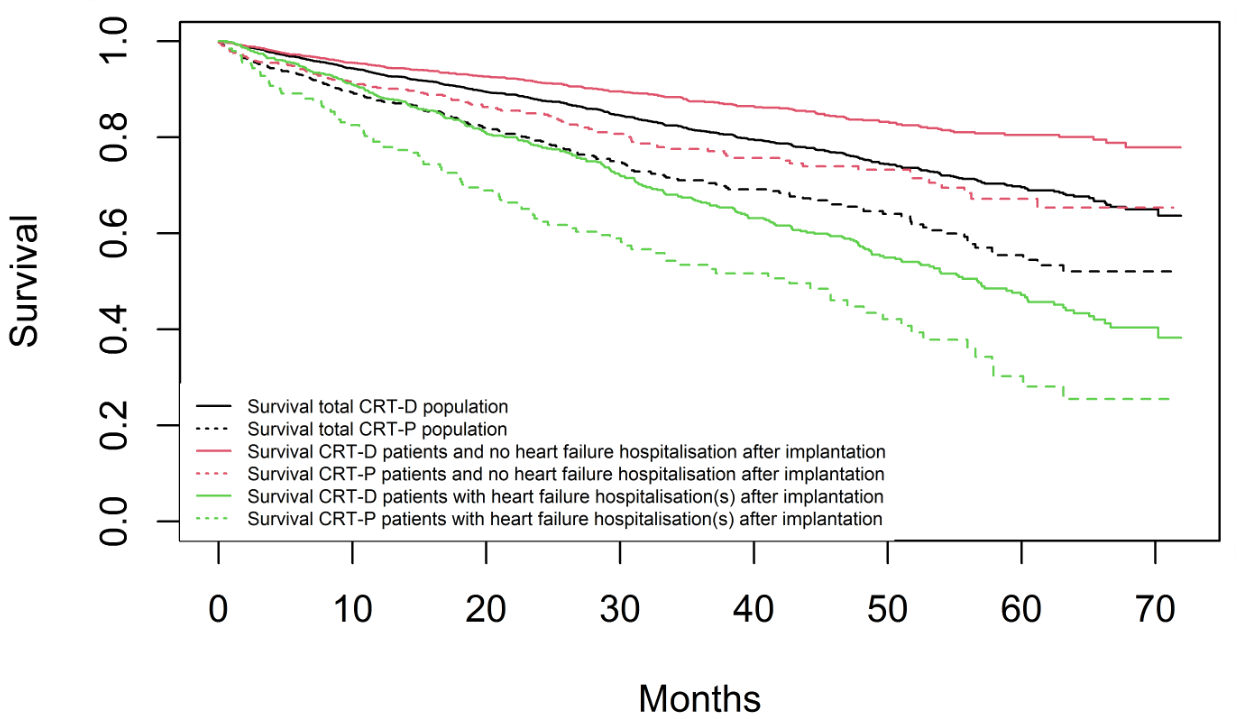


Figure SI1: Kaplan-Meier survival curves by heart failure hospitalisation after CRT implantation;
CRT = cardiac resynchronisation therapy, CRT-P = cardiac biventricular pacemaker; CRT-D = cardiac biventricular defibrillator (overall population N = 3,569)


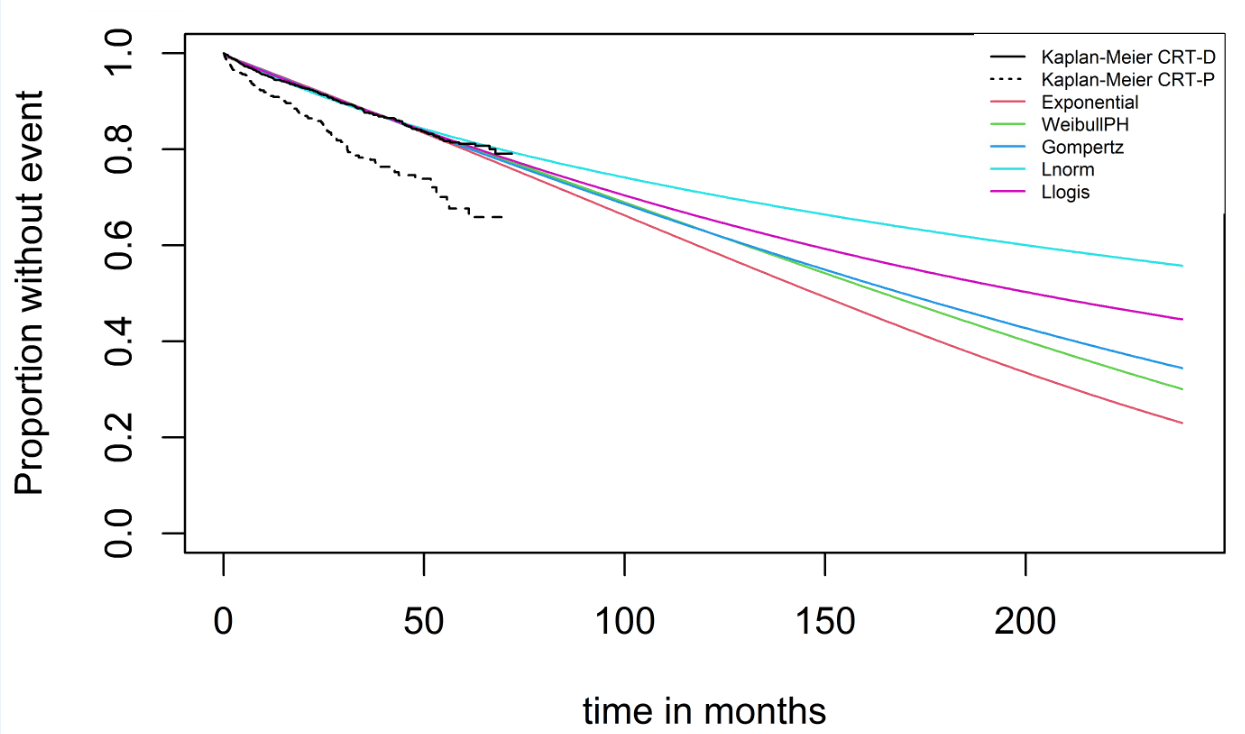


FigureSI2: CRT-D Kaplan-Meier curve for death in patients without any HF hospitalisation and estimated parametric survival curves for CRT-D patients for different distributions (n=2,726)


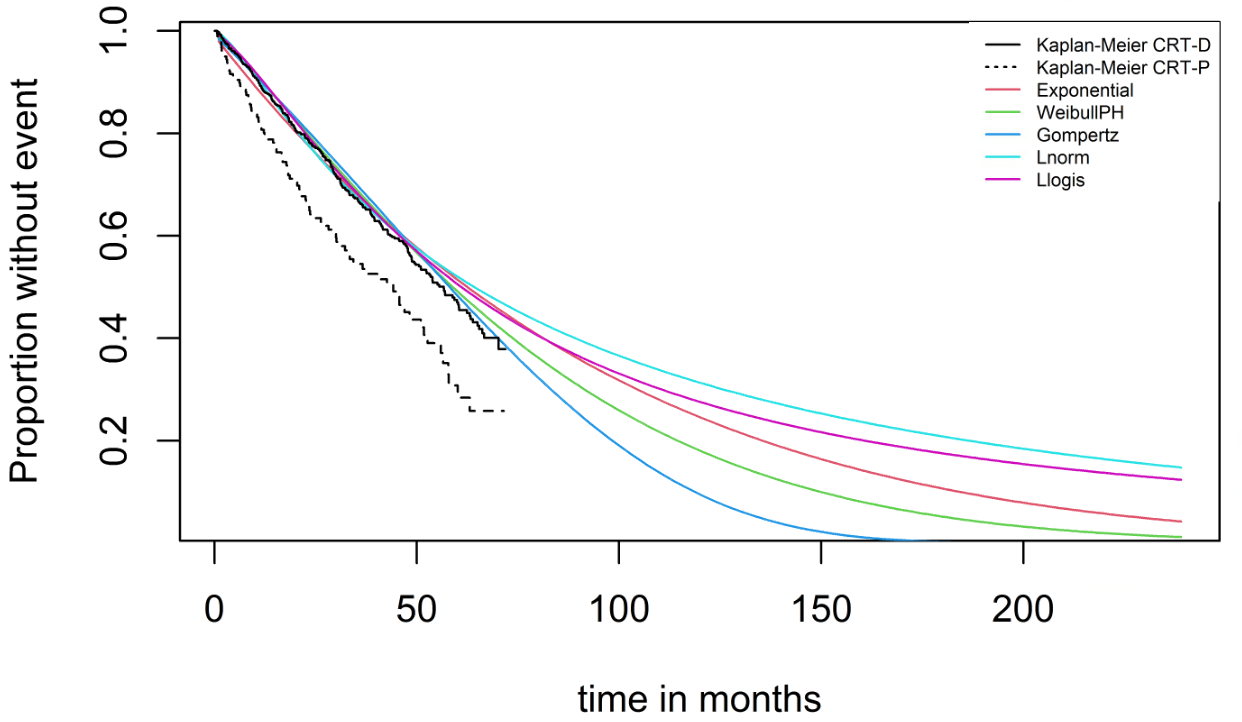


FigureSI3: CRT-D Kaplan-Meier curve for death in patients with one HF hospitalisation after CRT implantation and computed survival for CRT-D patients for different distributions (n=843)


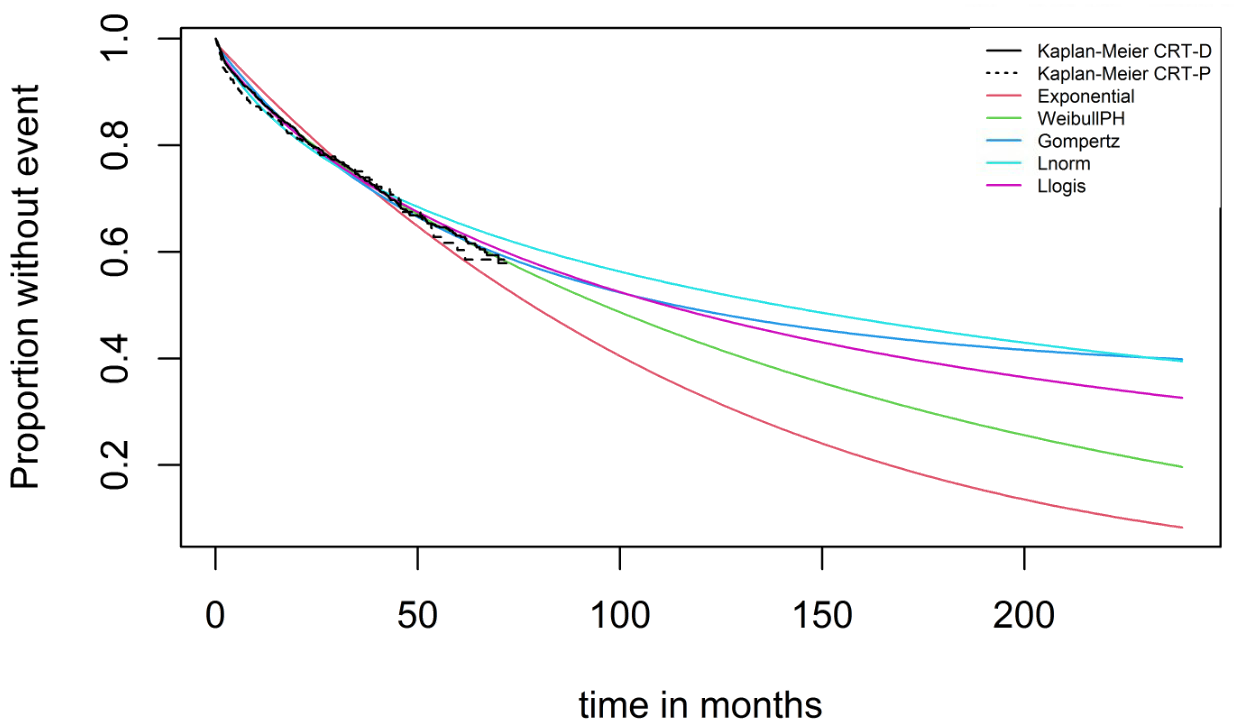


Figure SI4: CRT-D Kaplan-Meier curve for the first heart failure hospitalisation after CRT implantation and computed time for the first hospitalisation for CRT-D patients for different distributions (n=3,569)


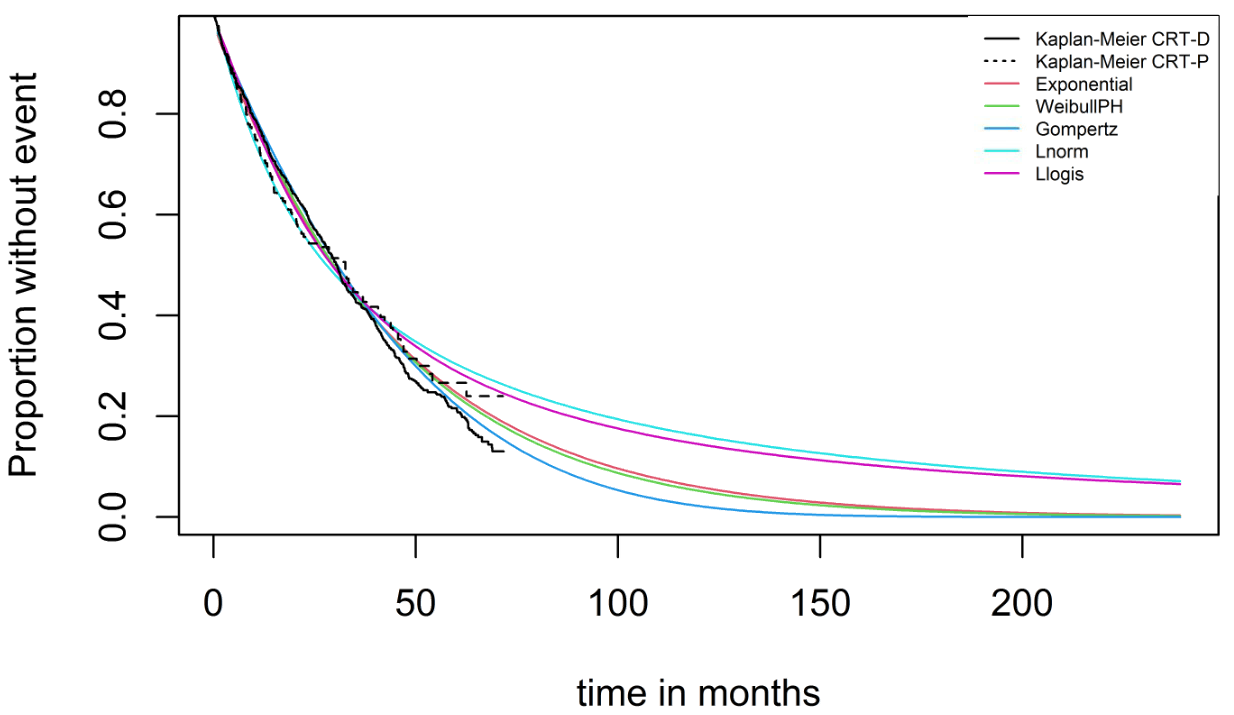


Figure SI5: CRT-D Kaplan-Meier curve for further heart failure hospitalisations after the first HF hospitalisation and computed time for further hospitalisation for CRT-D patients for different distributions (n=843)


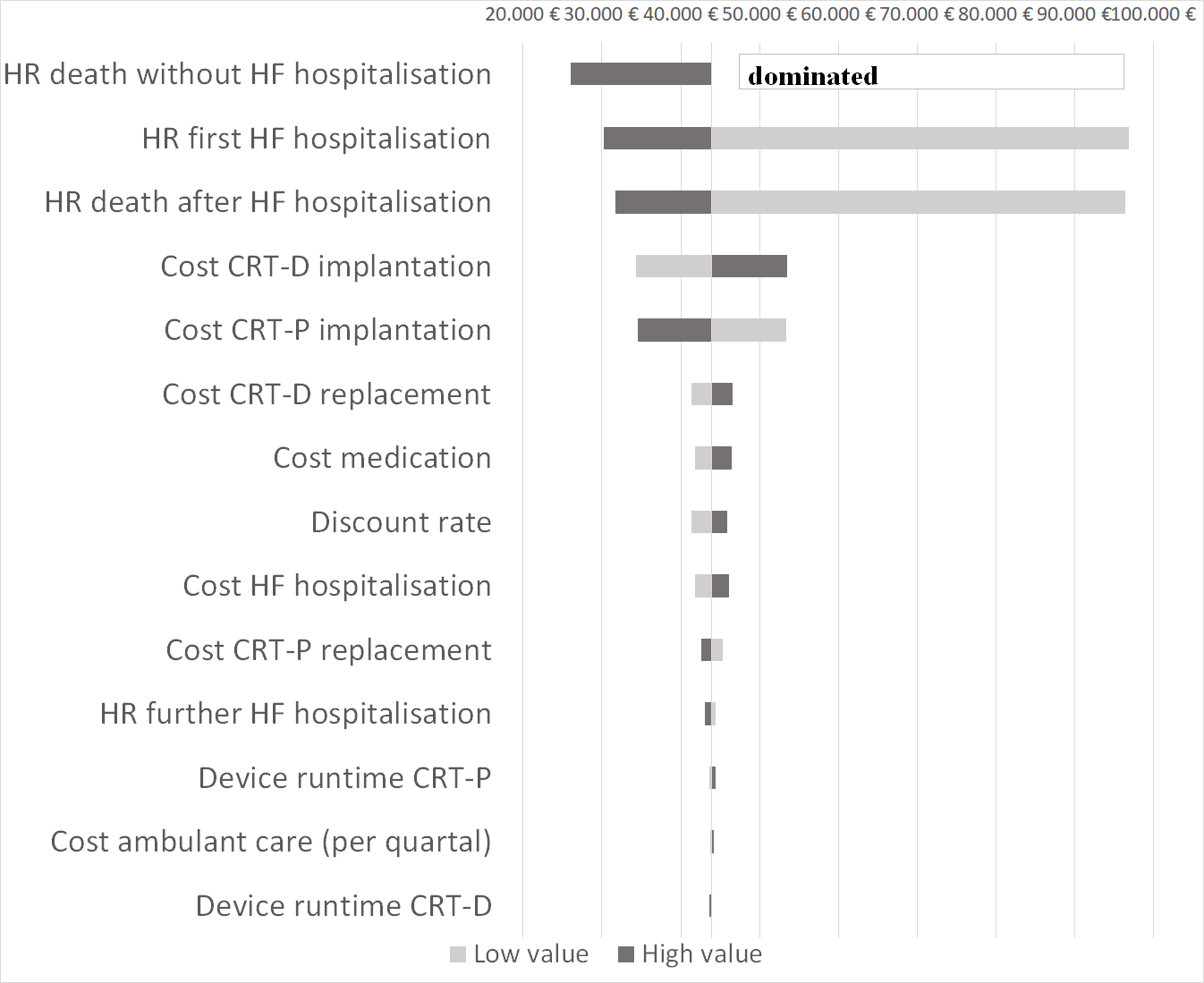


Figure SI6: Tornado chart

Abbreviations: CRT-P = cardiac biventricular pacemaker; CRT-D = cardiac biventricular defibrillator, HR = hazard ratio
